# Effect of elevated temperature on SARS-CoV-2 viability

**DOI:** 10.12688/f1000research.110305.2

**Published:** 2023-06-15

**Authors:** Harapan Harapan, Edison Johar, Chairin Nisa Maroef, Ida Yus Sriyani, Muhammad Iqhrammullah, Hendrix Indra Kusuma, Maimun Syukri, Razali Razali, Hamdani Hamdani, Rudi Kurniawan, Irwansyah Irwansyah, Sarwo Edhy Sofyan, Khin Saw Myint, T.M. Indra Mahlia, Samsul Rizal

**Affiliations:** 1 Department of Microbiology, School of Medicine, Universitas Syiah Kuala, Banda Aceh, 23111, Indonesia; 2 Tropical Disease Centre, School of Medicine, Universitas Syiah Kuala, Banda Aceh, 23111, Indonesia; 3 Medical Research Unit, School of Medicine, Universitas Syiah Kuala, Banda Aceh, 23111, Indonesia; 4 Eijkman Institute for Molecular Biology, Jakarta, 10430, Indonesia; 5 Graduate School of Mathematics and Applied Sciences, Universitas Syiah Kuala, Banda Aceh, 23111, Indonesia; 6 Department of Biology, Faculty of Mathematics and Natural Sciences, Universitas Syiah Kuala, Banda Aceh, 23111, Indonesia; 7 Department of Internal Medicine, School of Medicine, Universitas Syiah Kuala, Banda Aceh, 23111, Indonesia; 8 Department of Mechanical and Industrial Engineering, Faculty of Engineering, Universitas Syiah Kuala, Banda Aceh, 23111, Indonesia; 9 School of Civil and Environmental Engineering, University of Technology Sydney, Ultimo, Sydney, NSW 2007, Australia

**Keywords:** COVID-19, Isolation chamber, SARS-CoV-2, Temperature, Transmission

## Abstract

Severe acute respiratory syndrome coronavirus 2 (SARS-CoV-2) has caused a worldwide disruption of global health putting healthcare workers at high risk. To reduce the transmission of SARS-CoV-2, in particular during treating the patients, our team aims to develop an optimized isolation chamber. The present study was conducted to evaluate the role of temperature elevation against SARS-CoV-2 viability, where the information would be used to build the isolation chamber. 0.6 mL of the Indonesian isolate of SARS-CoV-2 strain 20201012747 (approximately 10 ^13^ PFU/mL) was incubated for one hour with a variation of temperatures: 25, 30, 35, 40, 45, 50, 55, 60, and 65°C in digital block heater as well as at room temperature (21-23°C) before used to infect Vero E6 cells. The viability was determined using a plaque assay. Our data found a significant reduction of the viral viability from 10 ^13^ PFU/mL to 10 ^9^ PFU/mL after the room temperature was increase to 40°C. Further elevation revealed that 55°C and above resulted in the total elimination of the viral viability. Increasing the temperature 40°C to reduce the SARS-CoV-2 survival could create mild hyperthermia conditions in a patient which could act as a thermotherapy. In addition, according to our findings, thermal sterilization of the vacant isolation chamber could be conducted by increasing the temperature to 55°C. In conclusion, elevating the temperature of the isolation chamber could be one of the main variables for developing an optimized isolation chamber for COVID-19 patients.

## Introduction

Coronavirus disease 2019 (COVID-19), caused by severe acute respiratory syndrome coronavirus 2 (SARS-CoV-2), has inflicted disruptions in many aspects of health systems globally. SARS-CoV-2 is an enveloped, non-segmented, positive sense, single-stranded RNA virus with a genome of approximately 30 kilobases. ^1^^,^^2^ The virus is mainly transmitted by nasopharyngeal droplets of an infected person; however, it could also be transmitted through aerosol. ^3^ The viability of the virus in the environment is influenced by several factors, including climatic parameters such as humidity and temperature. ^4^ Temperature has been proposed as a factor that affects SARS-CoV-2 transmission. ^5^ Previous studies found that the temperature effected the transmission of the SARS-CoV-2 in which high environmental temperature reduced the number of COVID-19 cases. ^6^^–^^8^ A study in State of Rio de Janeiro, Brazil found that the maximum and average of temperature were correlated negatively with COVID-19 infection. ^6^ Data from 117 countries also found a negative association between temperature and COVID-19 transmissibility in which an increase of 1°C could decrease COVID-19 prevalence by approximately 5.4%. ^7^ Several other investigations, however, have shown no evidence of a substantial influence of temperature on SARS-CoV-2 transmission. ^9^^,^^10^

Nevertheless data reveals that SARS-CoV-2 is highly susceptible to heat. ^11^ A recent study has shown that the virus could survive for at least 14 days at 4°C while only two days at 37°C and five minutes at 70°C. ^11^ Another study suggested that, at 40°C SARS-CoV-2-infected epithelial cells have reduced viral transcription and replication. ^12^ Although studies on the effect of elevated temperature on SARS-CoV-2 have been carried out, the temperature ranges used are limited. ^13^ In addition, the effect of temperature on viruses originating from Indonesia has not yet been published. As part of our project to optimize the isolation chamber for COVID-19 patients, we determined the effect of temperature on the resistance of SARS-CoV-2 originating from Indonesia by evaluating the viral viability with a range of temperatures from room temperature (21-23°C) to 65°C. Understanding viral survivability is critical for developing a temperature optimized isolation chamber that could minimize the risk of infection to healthcare workers and optimize energy consumption while ensuring comfort for patients. In addition, the information of this study might important for those who are living in the tropics.

## Methods

### SARS-CoV-2

SARS-CoV-2 strain 20201012747 isolated from Jakarta, Indonesia was used in this study. The virus originated from a patient with severe COVID-19 manifestation. The virus was kindly supplied by the Eijkman Institute for Molecular Biology. Before used in this study, the virus has been passaged twice on Vero E6 cells (ECACC, Vero C1008) (RRID: CVCL_0574). The virus was classified as an ancestral SARS-CoV-2 strain and isolated on 12 October 2020.

### Vero E6 cells

The Vero E6 cells were maintained in Modified Eagle Media (MEM) (Cat. no. 11090081) supplemented with 10% heat-inactivated fetal bovine serum (FBS) (Cat. no. 26140095), 3.5 mM Na _2_CO _3_ (Cat. no. 25080094), 1% penicillin-streptomycin-amphotericin B (Cat. no. 15240062), 25 mM 4-(2-hydroxyethyl)-1-piperazineethanesulfonic acid (HEPES) (Cat. no. 15630080), 1% non-essential amino acid (Cat. no. 11140050), and 2 mM L-glutamine (Cat. no. 25030081). All were from Gibco (Thermo Fisher Scientific, MA, USA). The cells were seeded into 12-well clear bottom plates (Cat. no. 3513, Corning, USA) for plaque assay.

### Exposure of temperatures

To expose to different temperatures, 0.6 mL of SARS-CoV-2 stock in 1.5 mL sterile tubes were incubated at room temperature RT (21-23°C), 25, 30, 35, 40, 45, 50, 55, 60, or 65°C for 1 hour in a digital block heater (Cat. no. 5382000031, Eppendorf, Germany). A separated experiment was conducted for each temperature; three replicates were used for each temperature and each replicate was repeated three times.

### Plaque assay

After the incubation at different temperatures, the viruses were diluted with a 10-fold serial dilution with 2% MEM (10 ^-1^ to 10 ^-12^) and 0.1 mL was inoculated onto 95-100% confluent monolayers of Vero E6 cells for 1 hour at 37°C with 5% CO _2_ with manual gentle shaking every 20 mins. After 1 hour of incubation, each well was then covered with 1 mL of 2% carboxymethyl cellulose (Cat. no. 17854-1KG, Merck, Germany) containing MEM, 2% FBS (Cat. no. 26140095), 3.5 mM Na _2_CO _3_ (Cat. no. 25080094), 25 mM HEPES (Cat. no. DMEM 15630080), 1% non-essential amino acid (Cat. no. 11140050), and 1% penicillin-streptomycin-amphotericin B (Cat. no. 15240062); All from Gibco (Thermo Fisher Scientific, MA, USA). The plates were incubated in cell incubator for 72 hours at 37°C and 5% CO _2_. The cells were then fixed with 4% paraformaldehyde for 4 hours at room temperature, and stained with 1% crystal violet. The plaques were counted manually. All works with infectious SARS-CoV-2 were conducted in the Biosafety Level 3 Laboratory at the Eijkman Institute for Molecular Biology in Jakarta. The plaque forming unit (PFU/mL) was calculated by dividing the number of plaques by dilution factor.

## Results

**Figure 1.**
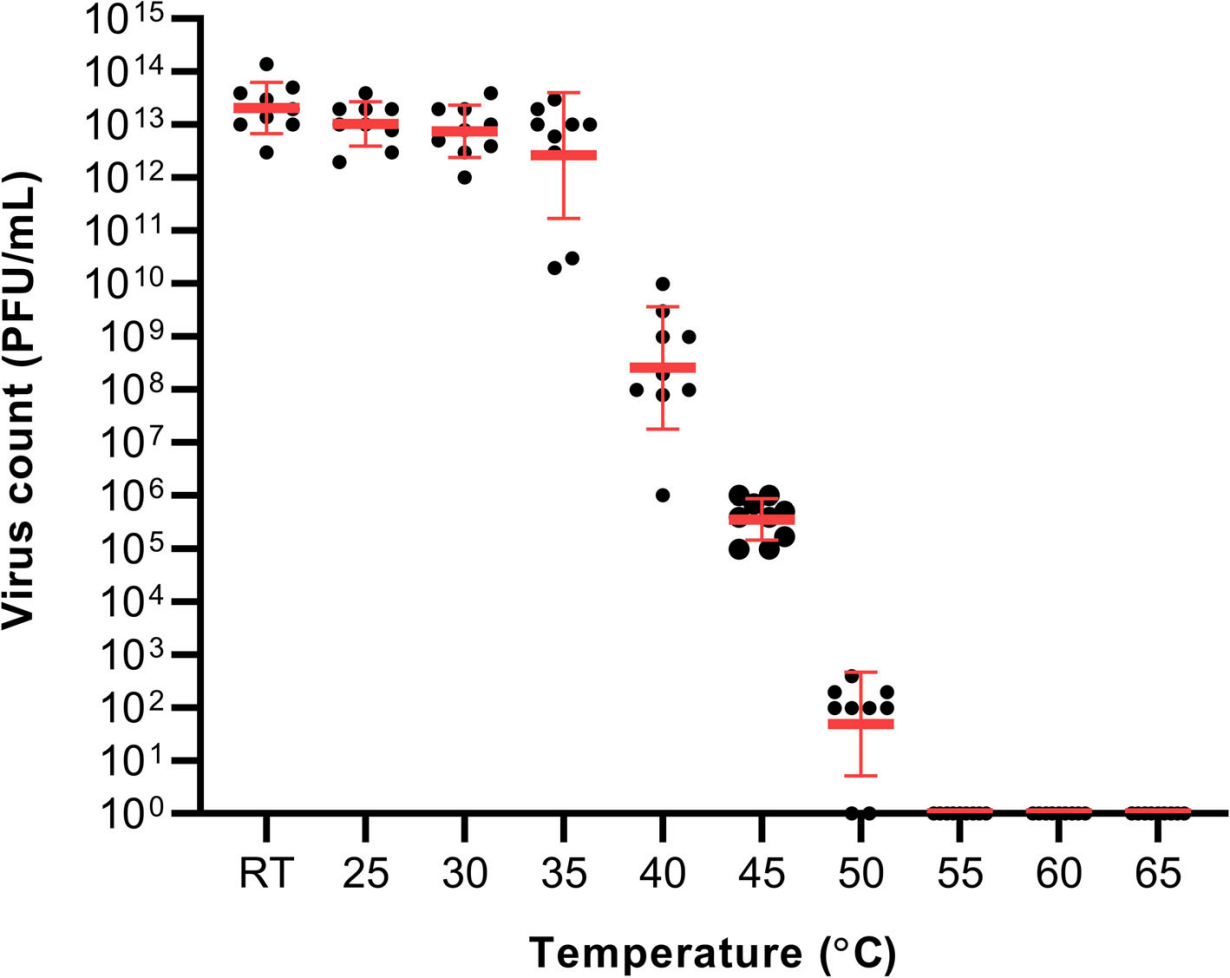
The effect of temperature on severe acute respiratory syndrome coronavirus 2 viability. RT – (21-23°C).

The virus was observed to remain stable from RT to 35°C with an average plaque count of 10 ^13^ PFU/mL. ^23^ A reduction in the viral count was observed in the 40°C treatment group at 10 ^9^ PFU/mL. Increasing the temperature to 45°C resulted in a further reduction of the viral viability resulting in 10 ^6^ PFU/mL. The last temperature with visible plaque was in the 50°C treatment group with a result of 10 ^2^ PFU/mL. The reduction of SARS-CoV-2 viability had a temperature-dependent trend within the temperature range of 35 to 50°C ( Figure 1). No plaques were visible in the 55, 60 and 65°C treatment groups.

## Discussion

Nosocomial transmission of SARS-CoV-2 has been identified to occur via multiple routes in healthcare facilities ^14^ indicating that uncomplicated measures like wearing personal protective equipment along with surface cleaning and decontamination ^15^ could be used effectively to reduce the transmission of infection. Other than that, modification to facilities such as, including isolation chambers with temperature control could help minimize the transmission. ^11^^,^^16^ As noted in a previous study, ^11^ temperature affects the stability of the virus in aerosol or on a surface. When the temperature of the room is not elevated, SARS-CoV-2 could remain stable for up to 72 hours on a surface such as plastic and stainless steel and three hours in aerosols. ^16^ Based on the findings of this present study, to reduce the viral viability significantly, a higher room temperature might be important. However, according to a previous report, the average maximum temperature for Indonesian people to still feel comfortable falls between 24 and 29°C ^17^ and to increase the room temperature would probably cause discomfort to the patient.

The administration of heat to the isolation chamber could be conducted prior to patient handling to reduce the likelihood of SARS-CoV-2 transmission. The other possibility is using heat in the isolation chamber as a means of thermotherapy. Thermotherapy is where mild-temperature elevation or hyperthermia (39-42°C) is used as a treatment against SARS-CoV-2 infection. ^18^ Previous studies assessing the indoor temperature and SARS-CoV-2 were associated inactivation of SARS-CoV-2. ^19^^–^^21^ Following the increase in temperature, heat-shock proteins (HSPs) are released which downregulates the progression of sepsis-induced acute lung injury. ^22^ However, HSPs could become hosts to several viruses (such as human papillomavirus, adenovirus, and dengue virus) promoting their infectivity. ^23^ In the case of SARS-CoV-2, its infectivity is more likely to be degraded than promoted by the HSPs. ^18^ Therefore, heat administration to the isolation chamber should not be performed on COVID-19 patients with human papillomavirus, adenovirus, or dengue virus co-infections. In addition, it should not be attempted on patients with severe-to-critical COVID-19 as they would be more likely to have an increased risk of mortality following the thermotherapy or heat administration. ^24^

One of the limitations of this study was we did not assess the role of other factors that might influence the SARS-CoV-2 transmission such as humidity. Therefore, the results of our study should be incorporated with data of the other studies assessing the effects of humidity on SARS-CoV-2 viability. In addition, we assessed the effect of the temperature on SARS-CoV-2 in a liquid state only and this might have influenced the results. Assessing the effect of the temperature on different states might could provide better understanding.

## Conclusions

This present study also has proven that increasing indoor temperature to 55°C is sufficient to terminate the virus. Further increment to the temperature would not be necessary and only results in higher energy consumption. Similarly, a previous study also reported the inactivation of 90% of SARS-CoV-2 achieved at 54.5°C after 36 minutes. ^13^ However, for use in treatment 55°C, might be too high for patients to tolerate. In that case, we only suggest the use of such temperature to thermally sterilize the isolation chamber prior to its use.

## Data availability

### Underlying data

Figshare: Effect of elevated temperature on SARS-CoV-2 viability. DOI: https://doi.org/10.6084/m9.figshare.19243515.v1. ^25^

This project contains the following underlying data:

‐ Master Table.xlsx [Table containing the raw data of the study]

Data are available under the terms of the Creative Commons Attribution 4.0 International license (CC-BY 4.0).

## Ethics statement

Not applicable.
