# Supplementary material for: Effect of elevated temperature on SARS-CoV-2 viability
Source: F1000Res. 2023 Jun 15;11:403. Originally published 2022 Apr 8. [Version 2] doi: 10.12688/f1000research.110305.2 (PMC10517306; doi:10.12688/f1000research.110305.2)
Supplement: Peer review history

# Reviewer report: Reviewer response for version 2

**DOI:** 10.5256/f1000research.144182.r194028

**Published:** 2023-09-22

**Authors:** Katherina Sewald

**Affiliations:** 1 Fraunhofer Institute for Toxicology and Experimental Medicine ITEM, Hanover, Germany

The authors addressed my questions and included limitations of their study into the discussion.

Is the work clearly and accurately presented and does it cite the current literature?

No

If applicable, is the statistical analysis and its interpretation appropriate?

Partly

Are all the source data underlying the results available to ensure full reproducibility?

No source data required

Is the study design appropriate and is the work technically sound?

Yes

Are the conclusions drawn adequately supported by the results?

No

Are sufficient details of methods and analysis provided to allow replication by others?

Partly

Reviewer Expertise:

Expertise in in vitro / ex vivo infection research, focus on respiratory diseases.

I confirm that I have read this submission and believe that I have an appropriate level of expertise to confirm that it is of an acceptable scientific standard.

# Reviewer report: Reviewer response for version 2

**DOI:** 10.5256/f1000research.144182.r193572

**Published:** 2023-08-24

**Authors:** Alex Wing Hong Chin, Chi Yeung Shuen

**Affiliations:** 1 School of Public Health, The University of Hong Kong, Hong Kong, Hong Kong; 2 Centre for Immunology & Infection, Shatin, Hong Kong

This study involves a single experiment to demonstrate the effect of elevated temperature on SARS-CoV-2 stability. The experimental setting and findings were very similar as the previously published report. One of the major purposes of the authors is to apply the findings to develop a temperature optimized isolation chamber in a hospital setting. However, I have reservation on the conclusion that elevating the temperature of an isolation chamber with COVID-19 patient will reduce the risk of transmission to the healthcare worker.

 On one hand, patient continuously shed virus to the environment. Provided that respiratory droplets and aerosols are the major routes of transmission, pre-heating the chamber for one hour before the healthcare worker coming in will have minimal effect on the continuous droplet and aerosol generation. If the purpose is to reduce the risk of fomite transmission, simple surface disinfection will be more efficient and safer. The authors provided evidence by other groups that report negative association between air temperature and COVID-19 transmission in different countries. However, these findings are controversial. Many other studies found no evidence on higher temperature will have negative impact on COVID-19 transmission and some even found the reverse.

 On the other hand, it may not be desirable to place a patient in an undesirable environment at 40 degree C, not to mention the increased risk of heat related illness, like heat exhaust or heat stroke, which can be lethal. I think this needs to be considered very carefully. In addition, it is mentioned in the abstract that increasing the environmental temperature to 40 degree C will create mild hyperthermia condition in patient and reduce SARS-CoV-2 survival. However, there is no evidence to support this claim. The viability of virus in culture medium without present of any cell may not be the same as in the respiratory track covered with epithelial cells at the same temperature.

 There are other flaws in this manuscript including the experimental design and data presentation. The viability of the virus was only studied in medium. Although the authors mentioned this as a limitation of the study, assessing of the viral stability in droplet / aerosol exposed to hot air would be more relevant and essential for coming to the current conclusion. Plaque assay is fine for determining the viral titre of infectious virus. However, the information on limit of detection of the assay is missing. No plaque observed in the assay does not necessarily mean that the viral titre is zero. The input control without one hour incubation was also missing, so one has no idea about the initial titre. Statistical analysis was also missing.

Is the work clearly and accurately presented and does it cite the current literature?

No

If applicable, is the statistical analysis and its interpretation appropriate?

No

Are all the source data underlying the results available to ensure full reproducibility?

No source data required

Is the study design appropriate and is the work technically sound?

No

Are the conclusions drawn adequately supported by the results?

No

Are sufficient details of methods and analysis provided to allow replication by others?

Partly

Reviewer Expertise:

Virology

We confirm that we have read this submission and believe that we have an appropriate level of expertise to state that we do not consider it to be of an acceptable scientific standard, for reasons outlined above.

# Reviewer report: Reviewer response for version 1

**DOI:** 10.5256/f1000research.121896.r137526

**Published:** 2022-06-30

**Authors:** Katherina Sewald

**Affiliations:** 1 Fraunhofer Institute for Toxicology and Experimental Medicine ITEM, Hanover, Germany

The authors addressed the research question whether elevated temperature has an effect on SARS-CoV-2 infectivity. The question might be important to establish conditions in e.g. isolation chamber which reduces the transmission of SARS-CoV-2. There are already data about the temperature sensitivity of SARS-CoV-2 available, suggesting that SARS-CoV-2 is susceptible to temperature changes, in particular to heat. This temperature sensitivity influences the virus’ ability to replicate. Systematic approaches to assess the temperature sensitivity in detail is so far missing. This gap was filled by the herein submitted work. In general, the manuscript is well written, easy to understand.

 The methods were adequate to answer the research question. Choosing the plaque assay for determination of infective virus particles is sufficient. Using PCR would add no further information. Any description for virus propagation is missing. As the process and purity might have an influence on the results, I would add that information. Temperature steps were chosen in 5 °C intervals. I wondered why more physiological temperature were not also chosen, such as 31°C, 34°C…(as representatives in tracheal temperature when breathing e.g. 20°C warm air). This was recently addressed by https://doi.org/10.1093/infdis/jiac264. The sensitivity of the virus to temperature was only tested in a liquid state. The question about the stability as virus aerosol remained unanswered. It is a pity that the authors did not compare different virus strains. They focused only on SARS-CoV-2 strain 20201012747 isolated from Jakarta, Indonesia. I found no information about the Statistics used. I also missed some recent literature found by literature search.

 In general, I was not really convinced by the hypothesis that lifting the ambient temperature around an infected patient for one hour to 40 degree would substantially reduce the risk of infection to health care workers. As infection progresses, any exhalation of patient’ breath would increase the virus load in the air again. It is also necessary to understand what breathing of 40 °C warm air means for the air temperature in the trachea and in the alveoli as it could be reduced to core body temperature again – meaning that it remains without any effect if we think of using that for treatment of patients. Other methods for lifting body temperature might be more effective. 

 It is hard to say whether the information has great importance for others as the information provided is not substantially new. Other researchers might be interested in reading the study, in particular researchers focusing on transmission of SARS-CoV-2. Overall, this is a very small publication showing little results. These results are not outstanding, in terms of the content of the results per se and not in terms of the methods/techniques used and also not in terms of the effort involved, which might also justify indexing.

 Minor point:

- M&M part: please change from two times to twice

Is the work clearly and accurately presented and does it cite the current literature?

No

If applicable, is the statistical analysis and its interpretation appropriate?

Partly

Are all the source data underlying the results available to ensure full reproducibility?

No source data required

Is the study design appropriate and is the work technically sound?

Yes

Are the conclusions drawn adequately supported by the results?

No

Are sufficient details of methods and analysis provided to allow replication by others?

Partly

Reviewer Expertise:

Expertise in in vitro / ex vivo infection research, focus on respiratory diseases.

I confirm that I have read this submission and believe that I have an appropriate level of expertise to confirm that it is of an acceptable scientific standard, however I have significant reservations, as outlined above.

## References

[1] : Differences in New Variant of Concern Replication at Physiological Temperatures In Vitro. *J Infect Dis* .2022; 10.1093/infdis/jiac264 10.1093/infdis/jiac264 35759271PMC9384407

# Reviewer report: Reviewer response for version 1

**DOI:** 10.5256/f1000research.121896.r134477

**Published:** 2022-04-13

**Authors:** Ramadhan Tosepu

**Affiliations:** 1 Deparment of Environmental Health, Faculty of Public Health, University of Halu Oleo, Kendari, Indonesia

Methods of preventing the spread of Covid-19 are continuously being studied, environmentally friendly methods have a positive impact on the community. The research is very good and useful for health workers and the public, so it deserves to be indexed. In the future, this research will be a reference for policy makers in dealing with COVID-19. 

**Introduction**

- Add that this research is useful for people living in the tropics. 

**Discussion**

- Temperature is not the sole cause of disease breading, so in this section it is necessary to add research limitations, such as humidity factor which are not included in this research study.
- It is advisable to add research related to indoor temperature.

**Conclusions**

- "This present study also has proven that increasing temperature" -> Indoor temperature

Is the work clearly and accurately presented and does it cite the current literature?

Yes

If applicable, is the statistical analysis and its interpretation appropriate?

Yes

Are all the source data underlying the results available to ensure full reproducibility?

Yes

Is the study design appropriate and is the work technically sound?

Yes

Are the conclusions drawn adequately supported by the results?

Yes

Are sufficient details of methods and analysis provided to allow replication by others?

Yes

Reviewer Expertise:

NA

I confirm that I have read this submission and believe that I have an appropriate level of expertise to confirm that it is of an acceptable scientific standard.
